# The impact of high‐grade complications on quality of life and psychosocial distress in the early period after radical cystectomy

**DOI:** 10.1002/bco2.70222

**Published:** 2026-06-11

**Authors:** Guido Müller, Henning Bahlburg, Mayumi Götte, Marius Cristian Butea‐Bocu, Florian Roghmann, Joachim Noldus

**Affiliations:** ^1^ Center for Urological Rehabilitation Kliniken Hartenstein Bad Wildungen Germany; ^2^ Department of Urology Marien Hospital Herne, Ruhr‐University Bochum Herne Germany

**Keywords:** bladder cancer, high‐grade complications, psychosocial distress, quality of life, radical cystectomy

## Abstract

**Objectives:**

This study aims to investigate the impact of high‐grade complications (i.e., Clavien‐Dindo Classification [CDC] Grade ≥III) on quality of life (QoL) and psychosocial distress (PD) in the early period after radical cystectomy (RC) and urinary diversion.

**Patients and Methods:**

The study relied on prospectively collected data of patients undergoing 3 weeks of inpatient rehabilitation (IR) after RC and urinary diversion (ileal conduit [IC] or ileal neobladder [INB]) between 04/2018 and 12/2019. Patients were surveyed on QoL (EORTC QLQ–C30) and PD (QSC–R10) by validated questionnaires at the beginning and the end of IR. Information about complications before the start of IR was taken from the hospital discharge letters and patient interview.

**Results:**

Overall, 842 patients were enrolled. High‐grade complications occurred in 25.5% of patients. Men (27.4% vs. 17.6%, *p* = 0.011) and patients with an INB (28.9% vs. 22.6%, *p* = 0.037) were significantly more susceptible to high‐grade complications. At the beginning of IR, health‐related QoL (HRQoL), role and social functioning were significantly lower in patients with a high‐grade complication. Both HRQoL and the proportion of patients with high PD improved significantly during IR (*p* < 0.001, respectively). A multivariable linear regression analysis identified high‐grade complications to significantly impact HRQoL (*p* = 0.013). Meanwhile, age (*p* = 0.001), INB (*p* = 0.019) and high‐grade complications (*p* = 0.01) significantly contributed to PD.

**Conclusion:**

High‐grade complications after RC and urinary diversion significantly impair short‐term QoL and PD, warranting constant physical and psychosocial monitoring.

## 1 INTRODUCTION

Radical cystectomy (RC) and urinary diversion is the gold standard for the treatment of localized muscle invasive bladder cancer (MIBC)^1^, ^2^ and may also be offered to patients with non‐muscle invasive bladder cancer (NMIBC).^3^, ^4^ Ileal conduit (IC) and ileal neobladder (INB) are the two most common types of urinary diversion.^5^, ^6^ RC is associated with a significant rate of both minor and major complications and mortality in the peri‐ and early post‐operative period.^7^, ^8^, ^9^, ^10^, ^11^ Our group recently highlighted that almost 97% of patients experience any type of complication after RC, with INB and higher Charlson Comorbidity Index (CCI) independently predicting a high‐grade complication (Clavien‐Dindo Classification [CDC] ≥ III^12^) in the first 6 weeks after RC.^13^ RC places a considerable burden on the patient and impairs numerous functional and psychosocial aspects of life. Quality of life (QoL) and psychosocial distress (PD) have each been widely studied both in the perioperative setting as well as long‐term after RC.^14^, ^15^, ^16^, ^17^, ^18^, ^19^ As expected, both outcomes are severely impacted by RC, but QoL recovers over time independent of type of urinary diversion.^15^ However, as recently demonstrated, >40% of patients suffer from elevated long‐term PD.^15^ To this day, the impact of complications on health‐related quality of life (HRQoL) and PD in the early period after RC and urinary diversion remains unclear. As demonstrated by Kretschmer et al., high‐grade complications led to reduced physical functioning levels 3 months after surgery, without impact on global HRQoL.^20^ One small prospective randomized single‐centre study in 50 Danish patients did not find a difference in QoL in patients with or without major complications after either open RC (ORC) or robot‐assisted RC (RARC),^21^ while PD was not assessed. Therefore, this study aims to fill this gap in knowledge of the impact of high‐grade complications on QoL and PD in the early period after RC by incorporating all measures into a comprehensive analysis.

## 2 PATIENTS AND METHODS

### 2.1 Patient cohort and outcome measurements

This prospective study is based on clinical data of patients with urothelial carcinoma of the bladder who underwent RC with creation of IC or INB in various hospitals across Germany and who were treated in a specialized centre for urological rehabilitation (Kliniken Hartenstein, Bad Wildungen, Germany) between April 2018 and December 2019. The study protocol was approved by an institutional research committee (research authorization number FF30/2017). For the purpose of this specific study, QoL and PD were measured by validated questionnaires (European Organization for Research and Treatment of Cancer [EORTC] QLQ–C30^22^ and Questionnaire on Stress in Cancer Patients [QSC]–R10,^23^ respectively) at the beginning and the end of inpatient rehabilitation (IR).

The 30 questions of the EORTC QLQ‐C30 can be answered on a graded scale (4‐point scale: *not at all*–*a little*–*moderately*–*very*; or 7‐point scale ranging from *very poor* to *excellent*). Shortness of breath, sleep disturbances, loss of appetite, constipation, diarrhoea and financial difficulties are assessed using six individual items. Using two to five different items, functional scales (physical function, role function, cognitive function, emotional function, social function) and symptom scales (fatigue, nausea, vomiting, pain) as well as health‐related quality of life (HRQoL) are assessed. By transforming the scale values, scores between 0 and 100 per scale can be obtained. High scores on the HRQoL scale and the functional scales correspond to a higher quality of life, while high scores on the symptom scales indicate a greater burden of symptoms.

The QSC–R10 consists of 10 questions phrased in everyday language and assesses various psychosocial stressors specifically in patients with cancer. The questionnaire assesses fatigue and weakness, current pain, dissatisfaction with one's own body, the ability to discuss emotional stress, fear of disease progression, the partner's empathy regarding the current situation, sleep disturbances, limitations in leisure activities (e.g., sports) and lack of information regarding the disease or treatment, as well as tension and nervousness. The response options range from *not applicable* (0 points) to *applies* and affects the patient *barely* (1 point) to *very strongly* (5 points). A total score of ≥15 indicates high psychosocial distress.

Results and potential changes during IR were interpreted in accordance with the current literature.^22^, ^23^

Information on postoperative complications before the start of IR were extracted from discharge papers and patient interview. Complications were graded by the established CDC, with high grade complications being defined as CDC ≥ III.^12^

### 2.2 Inpatient rehabilitation

German social laws entitle every cancer patient to rehabilitation measures, which aim to restore physical and psychological functioning and facilitate a reintegration into the work force after cancer treatment. Accordingly, the current national bladder cancer guideline recommends IR after RC.^24^ Over time, dedicated specialized IR centres have developed. The Center for Urological Rehabilitation at Kliniken Hartenstein in Bad Wildungen, Germany, is the largest of its kind by annual volume (>5000 patients treated annually) and offers unique expertise in urological rehabilitation. During IR, patients are treated by specialized physicians, nurses and physiotherapists regarding urinary continence and stoma care, respectively. Psychosocial interventions are carried out by physicians, nurses, physiotherapists, psychologists and social workers. The programme includes information on bladder cancer and aftercare, individual, group and couple psychotherapy, relaxation training and psychoeducation.

### 2.3 Statistical analysis

Descriptive statistics for categorical variables included frequencies and proportions, while for continuous variables, medians and interquartile ranges (IQR) or means and standard deviations (SD) were reported. Between‐group comparisons were analysed using the Mann–Whitney *U* test or chi‐square test (Pearson) as appropriate. The Wilcoxon test was used to compare changes in quantitative variables, while the chi‐square test (McNemar) was used to compare changes in proportions. Multivariable linear regression analyses were performed to identify the impact of major complications on QoL and PD outcomes. Significance was considered at *p* < 0.05. Data were analysed by SPSS version 29.0 (IBM, Chicago, IL, USA).

## 3 RESULTS

### 3.1 Patient cohort and complications

Overall, 842 patients (683 male, 159 female) treated in 135 different primary hospitals from all over Germany were included in this study. IR started at a median of 28 days (IQR 23–34) and ended with a median of 50 days (IQR 47–58) after surgery. Four hundred and forty‐seven patients (326 male, 121 female) received an IC, while 395 (357 male, 38 female) received an INB (Table 1). ORC and RARC were performed in 89.0% and 11.0% of patients, respectively. Female patients received an IC significantly more often than an INB (ratio 3:1; *p* < 0.001). Patients receiving an IC were significantly older (median 73 vs. 64 years, *p* < 0.001) and more often suffered from cardiovascular diseases (72.9% vs. 55.9%; *p* < 0.001) and diabetes mellitus (16.1% vs. 10.6%; *p* < 0.021). Overall, 25.5% of patients experienced a high‐grade complication until the start of IR (median 28 days after RC [IQR 23–34]), most commonly genitourinary complications (8.3%) and wound healing disorders (12.5%). Men (27.4% vs. 17.6%, *p* = 0.011) and patients with an INB (28.9% vs. 22.6%, *p* = 0.037) were significantly more susceptible to a high‐grade complication. Surgical approach did not influence the occurrence of overall and high‐grade complications, respectively. Hospital readmission rate during IR was 9.4%, requiring a median stay of 5 days (IQR 3–7). No patient died during IR.

**TABLE 1:** Baseline characteristics of 842 patients after radical cystectomy for bladder cancer.

Variable	Total	Conduit	Neobladder	*p* *
Patients, *n* (%)	842 (100.0)	447 (53.1)	395 (46.9)	
Age (years), median (IQR)	68 (62–75)	73 (67–78)	64 (58–69)	**<0.001**
Sex, *n* (%)				
Male	683 (81.1)	326 (72.9)	357 (90.4)	**<0.001**
Female	159 (18.9)	121 (27.1)	38 (9.6)	**<0.001**
Karnofsky performance status (%), median (IQR)	80 (70–80)	70 (70–80)	80 (70–80)	**0.021**
BMI (kg/m^2^), median (IQR)	25 (23–28)	25 (23–28)	25 (23–27)	0.211
Cardiovascular disease, *n* (%)	547 (65.0)	326 (72.9)	221 (55.9)	**<0.001**
Diabetes, *n* (%)	114 (13.5)	72 (16.1)	42 (10.6)	**0.021**
Neoadjuvant chemotherapy, *n* (%)	83 (9.9)	37 (8.3)	46 (11.6)	0.102
Method of surgery, *n* (%)				
Robot‐assisted cystectomy	93 (11.0)	46 (10.3)	47 (11.9)	0.458
Open cystectomy	749 (89.0)	401 (89.7)	348 (88.1)	0.458
Tumour stage, *n* (%)				
≤pT2	562 (66.7)	262 (58.6)	300 (75.9)	**<0.001**
≥pT3	280 (33.3)	185 (41.4)	95 (24.1)	**<0.001**
Lymph node positive, *n* (%)^a^	131 (16.1)	86 (19.9)	45 (11.8)	**0.002**
No. of lymph nodes removed, median (IQR)	17 (12–25)	17 (12–26)	17 (12–24)	0.765

*Note*: Bold font indicates significant results.

Abbreviations: BMI, body mass index; IQR, interquartile range.

^a^ Data available for 815 patients (conduit *n* = 433 and neobladder *n* = 382).

* Mann–Whitney *U* test or chi square test (Pearson) as appropriate.

### 3.2 Impact of high‐grade complications on QoL

Patients experiencing a high‐grade complication before the onset of IR reported significantly worse HRQoL (mean 36.8 vs. 41.3, *p* = 0.02), role functioning (mean 31.3 vs. 37.6, *p* = 0.024) and social functioning (mean 43.4 vs. 49.7, *p* = 0.021) than patients without high‐grade complications. Physical, emotional and cognitive functioning did not differ between patients with or without a high‐grade complication before IR, respectively (Table 2). HRQoL improved significantly during IR in both patients with (36.8 vs. 53.7, *p* < 0.001) and without (41.3 vs. 60.0, *p* < 0.001) high‐grade complications. A multivariable linear regression analysis identified high‐grade complications to significantly impact HRQoL at the beginning of IR (regression coefficient [*r*] = −4.495, 95% confidence interval [CI] −8.050 to −0.939, *p* = 0.013). Meanwhile, age, male sex, INB and tumour stage did not influence QoL at this point (Table 3).

**TABLE 2:** QLQ‐C30 functional scales 28 days (IQR 23–34) after radical cystectomy in patients with (a) versus without (b) high grade complications (CDC III–V).

Variable	a Mean (SD)	b Mean (SD)	*p* *
Global health status/quality of life (HRQoL)	36.8 (21.2)	41.3 (21.4)	**0.020**
Physical functioning	57.6 (22.1)	60.4 (23.7)	0.115
Role functioning	31.3 (35.6)	37.6 (36.2)	**0.024**
Emotional functioning	53.4 (27.6)	56.1 (28.5)	0.241
Cognitive functioning	71.5 (28.1)	75.0 (25.2)	0.224
Social functioning	43.4 (31.4)	49.7 (32.3)	**0.021**

*Note*: Bold font indicates significant results.

Abbreviations: CDC, Clavien‐Dindo Classification; HRQoL, health‐related quality of life; SD, standard deviation.

* Mann–Whitney *U* test.

**TABLE 3:** Multivariable linear regression analysis to identify predictors of HRQoL and psychosocial distress 28 days (IQR 23–34) after radical cystectomy.

HRQoL				
Variable	*t*	Regression coefficient	95% CI	*p*
Age	−0.231	−0.023	−0.222 to 0.175	0.818
Male sex	−0.668	−1.351	−5.321 to 2.618	0.504
Neobladder	0.404	0.742	−2.862 to 4.346	0.686
Tumour stage ≥ UICC III	−0.541	−0.863	−3.993 to 2.267	0.588
High grade complications (CDC ≥ III)	−2.482	−4.495	−8.050 to −0.939	**0.013**

PD				
Variable	*t*	Regression coefficient	95% CI	*p*
Age	−3.299	−0.152	−0.243 to −0.062	**0.001**
Male sex	−0.440	−0.404	−2.208 to 1.400	0.660
Neobladder	−2.342	−1.950	−3.584 to −0.315	**0.019**
Tumour stage ≥ UICC III	1.342	0.965	−0.446 to 2.376	0.180
High grade complications (CDC ≥ III)	2.584	2.098	0.504 to 3.691	**0.010**

*Note*: Bold font indicates significant results.

Abbreviations: CDC, Clavien‐Dindo Classification; CI, confidence interval; HRQoL, health‐related quality of life; IQR, interquartile range; PD, psychosocial distress; UICC, Union Internationale Contre le Cancer.

### 3.3 Impact of high‐grade complications on PD

PD was significantly elevated at the beginning of IR in patients with high‐grade complications compared to those without (median 16 [IQR 9–25] vs. 14 [IQR 8–22], *p* = 0.042). By the end of inpatient rehabilitation, the QSC–R10 score in patients with high‐grade complications improved to a median of 11.0 (IQR 5–18.5), *p* < 0.001. Individual psycho‐oncological counselling sessions during IR were utilized by 42.1% of patients with high‐grade complications. During the inpatient rehabilitation period, the proportion of patients with high‐grade complications experiencing high psychosocial distress decreased significantly from 53.8% to 37.9% (*p* < 0.001).

A multivariable linear regression analysis (Table 3) identified age (*r* = −0.152, 95% CI −0.243 to 0.062, *p* = 0.001) and INB (*r* = −1.950, 95% CI −3.584 to −0.315, *p* = 0.019) to positively contribute to PD at the beginning of IR, while patients with a high‐grade complication reported significantly worse PD (*r* = 2.098, 95% CI 0.504–3.691, *p* = 0.010).

## 4 DISCUSSION

In this prospective cohort of 842 patients from all over Germany and all levels of care, both QoL and PD are significantly impacted by the occurrence of high‐grade complications in the immediate to early period after RC and urinary diversion.

Our data therefore oppose previous results by Vejlgaard et al., which did not report a measurable impact on QoL by major complications after RC with IC.^21^ Abozaid et al. demonstrated in a mixed cohort of patients with an INB and an IC that a high‐grade complication was a significant predictor of a deterioration in the global HRQoL score 3 months after surgery. In contrast to the single‐center analysis of Kretschmer et al. (*n* = 121),^20^ our study showed that 4 weeks postoperatively, patients with high‐grade postoperative complications had reduced role functioning as well as reduced social functioning and a significantly reduced global HRQoL. Obviously, the time of survey and study setting play an important role when evaluating these outcomes.

In the literature, risk factors for high‐grade complications vary between increasing age, obesity, higher comorbidity and INB.^9^, ^10^, ^13^ In our study, surgical approach did not influence the occurrence of overall and high‐grade complications, respectively. Compared to open surgery, the robot‐assisted approach resulted in less blood loss, shorter hospital stays and fewer thromboembolic events.^25^ A systematic review of Katsimperis et al. found no statistically or clinically significant differences in terms of high‐grade complications in relation to the surgical approach.^26^ Three and six months after surgery, there was no difference in quality of life between robot‐assisted radical cystectomy and open radical cystectomy.^27^ The study of Mastroianni et al. highlighted less impairment of symptom scales (fatigue, nausea and vomiting, pain, appetite loss) of the EORTC‐QLQ‐C30 in patients 6, 12 and 24 months after robot‐assisted radical cystectomy.^28^ However, opposed to our study, none of the abovementioned studies investigated complications and their impact on QoL outcomes within the first weeks after RC.

Bladder cancer is one of the most expensive cancers to treat.^28^ Scilipoti et al. call for strategies to reduce the increasing financial burden on the health care system caused by bladder cancer.^29^ As demonstrated, our rehabilitation programme improves QoL and reduces PD. In Germany, IR is a well‐established cost‐effective solution that enables an early return to work, thereby reducing the financial burden of bladder cancer on both patients and social funds. Early psychosocial and psycho‐oncological interventions (such as those performed during IR) may alleviate the long‐term psychological burden after RC and thereby additionally reduce health care costs. Admittedly, less than 50% of patients experiencing high‐grade complications underwent individual psycho‐oncologic counselling in addition to routine group counselling sessions. As we reported in a previous study, >42% of patients suffer from elevated PD in the long term.^15^ Importantly, patients seeking individual psycho‐oncologic counselling during IR were significantly more likely to suffer from high PD. This shows that greater attention should be drawn to the possibility of individual psycho‐oncologic counselling during IR. To our knowledge, our study is the first study to put high‐grade complications and PD after RC and urinary diversion into context. As highlighted by our analyses, patients experiencing a high‐grade complication in the immediate period after RC report significantly elevated PD levels. The need for more intensified psycho‐oncologic screening and counselling is highlighted by the fact that a considerable number of these patients underwent individual psycho‐oncologic counselling when it was offered to them. By these means, the proportion of patients with high‐grade complications experiencing high PD decreased significantly from 53.8% to 37.9% (*p* < 0.001) during IR.

Our study is not without limitations. First, due to the study design, we could not collect pre‐operative data on QoL and PD. Second, we could not discern whether ERAS (Enhanced Recovery After Surgery)–protocols or pre‐habilitation programmes were followed in the referring hospitals. As recently highlighted by Guney et al, targeted pre‐habilitation before RC may improve QoL and reduce postoperative complications.^30^ Third, the surgical experience of the primary hospital or even single surgeons may have influenced the rate of complications as previously described^31^ with potential downstream effects on QoL and PD. Since patients from 135 primary hospitals were included and hospitals may refer their patients to different IR centres, surgical volumes can only be estimated. Furthermore, with regard to the described improvements in QoL and PD during the IR period, a control cohort outside of IR is missing. Based on many years of experience in treating patients after RC, it is assumed that almost all patients after RC undergo IR. However, data in this regard are lacking. Furthermore, QoL and PD outcomes were only assessed for this specific study and are not regularly obtained during IR. Additionally, questionnaires were not interpreted on an individual level on the day of completion, and thus, targeted interventions could not be carried out. Moreover, in our analyses, we do not differ between open surgery and robot‐assisted approach. Despite all listed limitations, our neutral third‐party assessment of the relationship of complications, QoL and PD paints a comprehensive picture of the inter‐connectiveness of physical and psychosocial outcomes after RC and urinary diversion in a large, prospective and homogeneous cohort. IR may help alleviate the impact of complications after RC on psychosocial outcomes, but since it is unique to the German healthcare system, comparative studies of QoL and PD across different healthcare systems are virtually impossible.

## 5 CONCLUSION

Patients experiencing a high‐grade complication after RC are at risk for short‐term impairment of both HRQoL and PD, which may be alleviated by a specialized rehabilitation programme. Further efforts are needed to reduce the rate of complications, for example, by a wider implementation of ERAS‐protocols or treatment in specialized high‐volume centres.

## AUTHOR CONTRIBUTIONS

**Guido Müller:** reviewing and editing the manuscript, project development, data collection, data analysis, other (funding acquisition). **Henning Bahlburg:** writing of the manuscript, data collection, data analysis. **Mayumi Götte:** review and editing the manuscript. **Marius Cristian Butea‐Bocu:** data collection. **Florian Roghmann:** reviewing and editing the manuscript. **Joachim Noldus:** project development, reviewing and editing the manuscript, other (funding acquisition).

## CONFLICT OF INTEREST STATEMENT

Regarding this research project, the authors have no relevant financial or non‐financial interests to disclose.

## CONSENT TO PARTICIPATE

All patients gave their informed consent prior to their inclusion in the study. All patients provided written consent to participate in this study.

## CONSENT TO PUBLISH

The authors affirm that all participants provided informed consent that data obtained in this study may be published.

## Data Availability

Data are not publicly available but can be made available by the corresponding author upon reasonable request.
